# Comparative mitochondrial proteomics: perspective in human diseases

**DOI:** 10.1186/1756-8722-5-11

**Published:** 2012-03-18

**Authors:** Yujie Jiang, Xin Wang

**Affiliations:** 1 Department of Hematology, Provincial Hospital affiliated to Shandong University, Jinan, China

**Keywords:** Mitochondrial proteome, Comparative proteomics, Mass spectrometry, Biomarkers

## Abstract

Mitochondria are the most complex and the most important organelles of eukaryotic cells, which are involved in many cellular processes, including energy metabolism, apoptosis, and aging. And mitochondria have been identified as the "hot spot" by researchers for exploring relevant associated dysfunctions in many fields. The emergence of comparative proteomics enables us to have a close look at the mitochondrial proteome in a comprehensive and effective manner under various conditions and cellular circumstances. Two-dimensional electrophoresis combined with mass spectrometry is still the most popular techniques to study comparative mitochondrial proteomics. Furthermore, many new techniques, such as ICAT, MudPIT, and SILAC, equip researchers with more flexibilities inselecting proper methods. This article also reviews the recent development of comparative mitochondrial proteomics on diverse human diseases. And the results of mitochondrial proteomics enhance a better understanding of the pathogenesis associated with mitochondria and provide promising therapeutic targets.

## Introduction

Mitochondria, which are mainly composed by proteins and lipids, are considered as the most complex and the most important organelles of eukaryotic cells. They not only play a leading role in the energy metabolism, but are also closely involved in many cellular processes. Furthermore, mitochondria have a manageable level of complexity as a consequence of their apparent prokaryotic ancestry. Their endosymbiotic origins have been well preserved in their double membrane structure, and they possess their own circular genome with mitochondria-specific transcription, translation, and protein assembly systems [1]. Based upon the human genome, there is estimated to be approximately 2000 to 2500 mitochondrial proteins [2], however, just over 600 have been identified at the protein level [3]. For this reason, mitochondria contain a great number of proteins that have yet to be identified and characterized.

Due to the fact that proteins are the carriers of biotic movement, the mitochondrial proteome is deemed as an ideal target for global proteome analysis. In the past, many effects of disease processes in which mitochondria are involved have been studied using classic biochemical methods [4]. However, these studies usually focus on only one particular protein, but not on the whole mitochondrial proteome. Recent developments in proteomics have allowed more in-depth studies of proteins. Proteomics is the large-scale study of all proteins in an organism and allowes a global insight into the abundance of protein expression, localization, and interaction. Combining genomics, mass spectrometry, and computation, it is possible to systematically identify the mammalian mitochondrial proteome. The proteome is often used to investigate the pathogenesis, cellular patterns, and functional correlations on protein levels in a non-biased manner [5]. This proteomic approach also allows the possibility for developing new candidate biomarkers for the diagnosis, staging and tracking of disease. Comparative proteomics is a subset of proteomics whose primary purpose is revolving around the following fields: the investigation of the pathogenesis and mechanism of a drug, the discovery of new targets for diagnosis and treatment, and the examination of the effects of environmental factors on soma and cells. Thus, many significant proteins have been identified from normal and abnormal individuals often under various states treated by some agents. Researchers have made tremendous efforts to rapidly obtain results to study the differentially expressed proteins in the subcellular organelle. By doing so, the diversity of proteins can be unmasked and reveal the subcellular location information. Therefore, owing to the significant roles and functions in the cell, mitochondria have become a research "hot spot" in subcellular proteomics. With these new techniques, a thorough investigation of comparative mitochondrial proteomics becomes more and more achievable. Mitochondrial proteomic profiles have been generated across multiple organs, including brain, heart, liver, and kidney [6-8]. This review presents a summary of progression of the mitochondrial proteome in various human diseases using comparative proteomic techniques reported in recent years. Future prospects and challenges for the mitochondrial proteome will also be discussed.

## Techniques in comparative proteomics of mitochondria (both gel-based and gel-free)

### Gel-Based Techniques

Two-dimensional gel electrophoresis (2-DE) combined with mass spectra is still the most popular gel-based proteomic technique for comparative proteomics nowadays and has matured significantly over the past decades [9]. The most frequently used method is termed "bottom-up proteomics," which is a strategy using mass spectrometry or tandem mass spectrometry (MS/MS) to analyze proteolytically digested proteins [10]. Peptide mass fingerprinting (PMF) of digested peptide fragments using matrix-assisted laser desorption/ionization time of flight (MALDI-TOF) is the preferred method for an initial protein identification after separation by 2-DE due to its high throughput and cost efficiency [11]. However, 2-DE has many shortcomings in separating certain protein classes, such as membrane proteins, high molecular weight (> 200 kDa) or small molecularweight proteins (< 10 kDa), and basic proteins, ect. The application of 2-DE to study the mitochondrial proteome has its owndisadvantages [12,13]. Two-dimensional fluorescence difference gel electrophoresis (2D-DIGE) is the development of 2-DE that was originally introduced by Minden [14]. It also allows for the direct comparison of the changes in protein abundance changes, which is less than 10% across samples simultaneously with a 95% statistical reliability coefficient without interference due to gel-to-gel variation [15]. Moreover, another technique named BNPAGE (blue native gel electrophoresis) invented by Shägger and Jagow, is specialized for separating intact membrane protein complexes [16]. It has been primarily used to separate and isolate the five multi-polypeptide complexes of the oxidative phosphorylation (OXPHOS) system and the recovery of all respiratory chain complexes are approaching the level of detection [17].

### Non-Gel Based Techniques: MudPIT and ICATs

Multi-dimensional protein identification technology (MudPIT) combines the resolving power of high performance liquid chromatography (HPLC) with the analytical capacity of tandem mass spectrometry (MS/MS). Using this method, a complex protein mixture is first digested with a protease resulting in an even more complex peptide mixture that is resolved by multidimensional HPLC. As the peptides are eluted off of the column, they are analyzed by mass spectrometry. Yates et al introduced this automated multidimensional protein identification technology termed "shotgun" proteomics and demonstrated that a dynamic range of 10,000 to 1 between the most abundant and least abundant proteins/peptides in a complex peptide mixture could be identified [18]. MudPIT overcomes the shortcomings of mass spectrometry such as deficiencies in detecting proteins with extreme alkalinity, hydrophobicity, and maximum or minimum molecular mass. However, MudPIT cannot yet accomplish absolute quantitation. Gygi introduced isotope coded affinity tags (ICAT) in which isotypical and different biotin-containing moieties are conjugated to cysteine residues from two different peptide samples to quantitate the mixture of proteins [19]. This technique has been applied successfully to the detection of membrane proteins [20]. Shotgun proteomics combined with stable isotope labeling or label-free methods are effective in achieving absolute or relative protein quantification [21].

Many new proteomics techniques have been developed, such as iTRAQ (multiplexed isobaric tagging technology), protein chip, SELDI-TOF-MS (surface enhanced laser desorption/ionization of flight mass spectrometry), and SILAC (stable isotope labeling by amino acids in cell culture) [22-24]. Ultimately, the field of proteomics, with its depth and fast pace of investigation, has a tendency to combine multiple techniques so as to best utilize the benefits of each technique.

## Perspective on human disease and mitochondrial comparative proteomics

Applications of mitochondrial proteomics have shed some light on the diagnosis and treatment of many diseases associated with mitochondria. In addition, comparison of mitochondrial proteome from healthy and diseased tissues could result in the identification of biomarkers for the early diagnosis and pathologies concerned with mitochondrial dysfunction (Table 1).

**Table 1:** Overview of diseases associated with the mitochondrial proteome

Organ	Disease (Researcher)	Analytical method	Major proteins identified		Functional distribution	Primary Significance
					
			Up-regulated	Down-regulated		
***Nervous system***						

	**Alzheimer's disease (AD) **(Lovell MA.)	ICAT, 2D-LC/MS/MS	ATP synthase alpha chain		OXPHOS	Cells undergoing Aß -mediated apoptosis increase synthesis of proteins essential for ATP production and efflux to maintain metabolic functions.
	
			Pyruvate kinase, M1 isozyme		glycolysis	
	
			Glyceraldehyde 3 phosphate dehydrogenase		energy production	
	
			Cofilin		control of actin polymerization/depolymerization	
	
			Na+/K + -transporting ATPase a-3 chain		ATP production	
	
			VDAC 1 and 3		apoptosis	
	
			Dihydropyrimidinase- related protein-1 (DRP-1)		axon guidance, invasive growth and cell migration	

	**Multiple sclerosis (MS) **(Broadwater L.)	SELDI-TOF-MS		Cytochrome c oxidase subunit 5b (COX5b)	component of Complex IV of the electron transport chain	Proteins identified would be used as neuroprotective therapeutic targets for MS.
	
			Hemoglobin β-chain		oxygen transport	
	
			Myelin basic protein (MBP)		component of the myelin membrane in the CNS	

			Creatine kinase (CKB)		creatine metabolic process	

	**Neural degeneration **(Pienaar IS.)	2DE, ESI-QUADTOF/MS		Protein disulfide isomerase (PDI)	folding	Alteration of mitochondrial function may contribute to the beneficial effects associated with statin use.
	
			Heat shock proteins		protein assembly and folding	
	
				Dehydrogenase antiporter	transportation	
	
			Alpha-internexin (NF66)		cell differentiation, morphogenesis of neurons	
	
			Protein-tyrosine receptor type F polypeptide interacting protein (PTPRF)		cell adhesion receptor	
	
				Neuronal-specific enolase (NSE)	energy metabolism	
	
			Variation in ATP synthase, D chain		energy metabolism	
	
			Alpha-enolase-1 (ENO1)		glycolysis, growth control, hypoxia tolerance and allergic responses	
	
			Guanine nucleotide-binding proteins (G-proteins)		signal transduction	

***Cardiovascular system***						

	**Ischemia-induced cardiac injury **(Kim N.)	2-DE, MALDI-TOF-MS		Prohibitin	cell cycle	Proteomic analysis provides appropriate means for identifying cardiac markers for detection of ischemia-induced cardiac injury.

				VDAC	apoptosis	

	**Contractile dysfunction **(Essop MF.)	2D-PAGE, ESI-Q-TOF		ATP synthase D chain	OXPHOS	Decreased contractile protein levels may contribute to the contractile dysfunction of hearts from diabetic mice.
	
			Ubiquinol cytochrome-C reductase core protein 1		electron transport	
	
				Electron transfer flavoprotein subunit α	electron transport	

***Liver disease***						

	**Acetaminophen (APAP) affected the liver **(Ruepp SU.)	2D-DIGE, MALDI-TOF-MS		HSP10 and HSP60	protein assembly and folding	APAP toxicity was a direct action of its known reactive metabolite NAPQI, rather than a consequence of gene regulation.
	
			Heat shock 70 kDa protein 9, (GRP75)		transporters and channels	

	**High-fat diet induces hepatic steatosis **(Eccleston HB.)	2D-IEF/SDS-PAGE	uMUP-VIII major urinary protein		pheromone communication (only in rodents)	HFD causes steatosis, alters NO metabolism, and modifies the liver mitochondrial proteome, thus, NO may play an important role in the processes responsible for NAFLD.

			Thiosulfate sulfurtransferase		cyanide detoxification, role in iron-sulfur centers, sulfane metabolism	

				3-hydroxy-3-methylglutaryl-CoA synthase 2 (HMG-CoA synthase)	catalyzes the condensation of acetoacetyl CoA and acetone step in ketogenesis	

				Succinate dehydrogenase subunit a (SDH-A)	catalyzes the oxidation of succinate to fumarate, flavoprotein	

				ATP synthase F1 α and β subunits	OXPHOS	

***Skeletal muscl****e*						

	**Hypoxia-induced changes in rat skeletal muscle **(De Palma S.)	2D-DIGE, HPLC ESI-MS/MS	Hypoxia inducible factor 1 (HIF-1R)		transcription	In vivo adaptation to hypoxia requires an active metabolic switch.
	
			Pyruvate dehydrogenase kinase 1 (PDK1)		regulation of glucose metabolism	
	
				Mitochondrial dihydrolipoamide dehydrogenase	branched chain family amino acid catabolic process	
	
				Succinyl CoA ligase α chain	tricarboxylic acid cycle	

	**Lifestyle on the aging alterations (**Alves RM.)	2-DE, MALDI-TOF/TOF	NADH dehydrogenase [ubiquinone] 1 α subcomplex subunit 4		oxidative phosphorylation	Lifestyle is a key modulator for preventing aging-induced protein expression and functionality in mitochondria.

			Creatine kinase		signal transduction	

			Superoxide dismutase [Mn], mitochondrial		redox	

### Nervous System

Because the brain is considerably complex and hclewglnouo caccine organism, the orthodox empirical methods cannot meet the need to investigate the brain's constitution and function. The complexity of the nervous system is represented by the cellular categories and the number of synapses. Moreover, because the brain is a vital organ with massive energy consumption and can only utilize the energy produced from the process of anaerobic glycolysis, the role of mitochondriais very considerable in this tissue.

A series of studies have identified an abundance of alterations of in mitochondrial protein levels. To demonstrate that mutations in mitochondrial tRNA would affect the pattern of mitochondrial proteins, Rabilloud et al found a number of proteins in sibling hybrid cell lines using proteomic methods [25]. Two proteins that exhibited obvious large quantitative decreases were identified as nuclear-encoded subunits of cytochrome c oxidase. This finding clearly demonstrated a linkage between the effects of mutations in mitochondrial tRNA genes and the steady-state level of nuclear-encoded proteins in mitochondria. Alzheimer's disease (AD) is a fatal progressive neurodegenerative disorder whose etiology is unkown until now. Mitochondria may play a crucial role in the pathogenesis of AD. Chou and his colleagues analyzed the differential mitochondrial protein profile in the cerebral cortices of 6-month-old male 3 × Tg-AD (which harbor mutations in three human transgenes) and non-transgenic mice. Certain proteins which involved in a wide variety of metabolic pathways, such as the citric acid cycle, oxidative phosphorylation, pyruvate metabolism, and glycolysis, ect, were dysregulated in 3 × Tg-AD cortices. Interestingly, these alterations in the mitochondrial proteome occurred before the development of significant amyloid plaques and neurofibrillary tangles, indicating that mitochondrial dysregulation is an early event in AD [26]. In addition, the potential role of amyloid beta peptide (Aß)-mediated cell death in AD has been extensively investigated both in transgenic animal models and in neuron culture models. Lovell et al quantitatively measured changes in mitochondrial proteins of primary rat cortical neuron cultures exposed to Aß [27]. Ten proteins that were significantly altered in Aß-treated cultures were identified, including sodium/potassium-transporting ATPase, cofilin, dihydropyrimidinase, pyruvate kinase and voltage-dependent anion channel 1 (VDAC1). Elevations in the levels of proteins associated with energy production indicated that cells undergoing Aß-mediated apoptosis increased the synthesis of proteins essential for ATP production and efflux in an attempt to maintain metabolic function. Another similar study with Aß was reported by Gillardon [28]. They analyzed proteome changes in synaptosomal fractions from Tg2576 mice that over-express mutant human amyloid precursor protein (K670N, M671L) and from their non-transgenic littermates. Altered expression of certain proteins, such as heat shock protein 70 and changes in the subunit composition of the respiratory chain complexes I and III were identified. They concluded that mitochondria are early targets of Aß aggregates, and that elevated Aß might impair mitochondrial functions, thus providing a self-amylifying toxic mechanism. Another comparative proteomic investigation about neurodegenerative diseases was reported by Fu [29]. The expression of mitochondrial proteins involved in mitochondrial membrane potential, ATP production, and neuronal cell death was down-regulated after treatment of cerebellar granule neurons with bis(7)-tacrine. Thus, bis(7)-tacrine might be a beneficial agent for the treatment of neurodegenerative diseases. Pienaar and coworkers conducted a behavioral and quantitative mitochondrial proteomic analysis on the effects of simvastatin on a rat model of neural degeneration. Twenty-four mitochondrial proteins were identified in relative abundance after simvastatin treatment. Then they validated whether simvastatin was capable of altering sensorimotor function in a mitochondrial toxin-induced animal model. Rats were pre-treated with simvastatin for 14 days followed by a single unihemispheric injection of rotenone(a mitochondrial complex I inhibitor). The results showed that simvastatin improved motor performance in rotenone-infused rats. The results of behavioral and quantitative proteomic analysis are consistent and further exploration of these changes may identify promising bio-targets for degenerative disorders [30].

Multiple sclerosis (MS) is an inflammatory neurodegenerative disease of the central nervous system that results in progressive physical and cognitive disability. Broadwater et al utilized SELDI-TOF-MS to characterize the mitochondrial proteome in postmortem MS and control cortex. Peptide fingerprint mapping unambiguously identified four proteins, including cytochrome c oxidase subunit 5b (COX5b), the brain specific isozyme of creatine kinase, hemoglobin β-chain, and myelin basic protein (MBP), that could be used as neuroprotective therapeutic targets for MS [31].

As a whole, studies on the mitochondrial proteome of the nervous system provide a broader insight on various fractions of brain and the same fractions under various physiological and pathological states. However, most of the investigations are currently based on animal models because of the difficulty to obtain brain samples from humans. The results from the proteomics studies revealed that mitochondrial structural and functional alterations appear to play an important role in nervous system diseases.

### Cardiovascular System

Cardiovascular diseases have been the main "killer" in human beings, and thus, early diagnosis and treatment is imperative. Mitochondria are the major site of substrate oxidation in cardiomyocytes. Furthermore, oxidative stress plays a key role in heart diseases, and mitochondria are considered a principle source and target of reactive oxygen species (ROS) [32]. ROS can damage cellular lipids, proteins, and DNA, thereby disrupting their normal functions. Several large-scale studies have systematically reported some notable biological and medical insights into the mitochondrial proteome in the cardiovascular system as described below.

The ischemic heart is an important model for researchers studying the cardiovascular diseases. Until now, most of the findings from comparative mitochondrial proteomic studies were associated with the respiratory chains and energy metabolism in the ischemic heart. For example, Kim et al detected regional differences in protein expression levels from mitochondrial fractions of control, ischemia-reperfusion (IR), and ischemic preconditioned (IPC) rabbit hearts [8]. In addition, Essop and colleagues investigated the alterations in the mitochondrial proteome in a mouse model of obesity/type 2 diabetes. Several proteins that play role in mitochondrial energy metabolism, including ATP synthase D chain, ubiquinol cytochrome-C reductase core protein 1, and electron transfer flavoprotein subunit alpha, were identified to have changes in protein levels [33].

Mitochondria play a crucial role in the regeneration of antioxidants through the production of reducing equivalents and are responsible for the vast majority of ATP production within most cells and higher organisms. Hunzinger [34] employed a proteomics approach to investigate the role of ROS on bovine heart and identified two specific N-formylkynurenine modifications of aconitase-2, which is an enzyme that plays an important role in mitochondrial aging. Additional investigations on these two modifications might identify them as potential protein biomarker signatures for ROS. Serious and/or long-term ischemia will lead to heart infarction. It has been proposed that in ischemic preconditioning (PC) or pharmacological preconditioning, the GSK-3 (glycogen synthase kinase-3) inhibitor AR-A014418 will initiate signaling cascades that converges on mitochondria and results in cardioprotection. Therefore, Wong et al utilized 2D-DIGE coupled with Blue Native-PAGE to confirm their hypothesis that PC and pharmacological preconditioning similarly altered mitochondrial signaling complexes III, IV, and V. The altered expression levels of electron transport complexes obtained from the above-mentioned study should impart important implications for the mechanism of cardioprotection [35].

In short, most of the mitochondrial proteomics studies in cardiovascular diseases were associated with ROS. It is well known that ischemia and aging are the main causes of cardiovascular diseases, which can result in the release of ROS. Therefore, ROS are still a primary focus when researchers investigate cardiovascular disease. Targeting on ROS might be applicable to the treatment of many cardiovascular problems in the future.

### Cancer and Hematological Diseases

The mitochondrial proteome has altered expression levels and structures in cancer cells, as well as that in cells altered simulide when stimulated by various treatments (Table 2).

**Table 2:** Progress in the treatment of hematologic diseases and solid tumor based on mammalian mitochondrial proteomics

Disease	Cell lines/Treatment	Analytical methods	Up-regulated proteins	Down-regulated proteins	Functions	Primary significance
**Hematologic diseases**						

***AML***	NB4/camptothecin	2-DE, MALDI-TOF/TOF		Far upstream element-binding protein-1 (FUBP1)	transcription, translation, and degradation of proteins	Provided new insights for systematically understanding the mechanisms of the camptothecin-induced apoptosis.
	
				Heterogeneous nuclear ribonucleoprotein A1 (HNRPA1)	mRNA processing	
	
				Heterogeneous nuclear ribonucleoproteins C1/C2 (HNRPC)	modified with shift of p*I *and MW	
	
			26S protease regulatory subunit 6A (PSMC3)		degradation	
	
			Proteasome subunit alpha type (PSMA)-1, 2, 6		degradation	

***Non-Hodgkin's lymphoma***	Raji/Adramycin	DIGE, LTQ-ESI-MS/MS	ATP synthase d chain, mitochondrial (ATPQ, ATP5H)		OXPHOS	Specific mitochondrial proteins were uniquely susceptible to alterations in abundance following exposure to ADR and carry implications for the investigation of therapeutic and prognostic markers.
	
			Prohibitin (PHB)		cell cycle	
	
			Heat shock 70 kDa protein 9 precursor (HSPA9)		transporters and channels	
	
				Isoform 4 of Mitochondrial ATP-binding cassette sub-family B member 6 (ABCB6)	protein synthesis and degradation	
	
				Superoxide dismutase [Mn], mitochondrial precursor (SOD2)	redox	

**Other solid tumors**						

***Osteosarcoma***	143B/devoid of mitochondrial DNA	2-DE, MALDI-TOF/MS		NADH-ubiquinone oxidoreductase 75 kDa subunit	respiratory complexes subunits	Demonstrates the pleiotropic effects of mtDNA alterations and also gives valuable markers for the study of the mitochondrial-cytosolic coordination.

				Mitochondrial 28S ribosomal protein S2	mitochondrial translation apparatus	

				Mitochondrial import inner membrane translocase subunit Tim9	protein transport	

				Succinyl-CoA ligase (ADP-forming) beta-chain	energy production	

***Breast cancer***						

	MCF-7/resistance to adriamycin accompanied by verapamil	2-DE, QqTOF-ESI-MS/MS		Cofilin	control of polymerization/depolymerization of actin	Implications of the changes are considered with respect to drug resistance.
	
			CoproporphyrinogenIII(CPO)		heme biosynthesis	
	
			3.2 trans-enoyl CoA isomerase		fatty acid oxidation	
	
			Adenylate kinase 2		ATP, OXPHOS	

***Renal cell carcinomas***						

	UMRC2, 786-0 and RCC4/VHL (von Hippel Lindau)-defective	2-DE, MS	Heat shock 70 kDa protein		transporters and channels	Increased expression of septin 2 is a common event in RCC and protein modification may also alter septin 2 function in a subset of tumors.

			10-formyltetrahydrofolate dehydrogenase		one-carbon metabolism	

			Phosphoribosylglycinamide formyltransferase		purine biosynthesis	

			Ubiquinol cytochrome c reductase complex core protein 2		electron transport	

				Elongation factor 2	protein biosynthesis	

				Phosphofructokinase isozyme C	glycolysis	

				Thioredoxin reductase	differentiation, electron transport	

				Septin 2	cell cycle	

***Prostate cancer***						

	LNCaP/somatostatin		VDAC1, VDAC2		apoptosis	Somatos might be able to curb the progression of advanced prostate cancer.
	
				Peroxiredoxin 2 (PRDX2)	antioxidant activity	
	
				Translationally controlled tumor protein (TCTP)	calcium binding and microtubule stabilization.	

Chevallet et al [36] employed a comparative study on the osteoscrcoma 143B cell line and its Rho-0 counterpart (devoid of mitochondrial DNA). Quantitative differences were found between these cell lines in factors, such as the respiratory complexes subunits, the mitochondrial translation apparatus, mitochondrial ribosomal proteins, and the proteins with roles in the ion and protein import system. They also found that proteins involved in apoptosis control and import systems were differentially regulated in Rho-0 mitochondria. To identify proteins involved in a retrograde response and their potential role in tumorigenesis, Kulawiec [37] conducted a comparative proteomic analysis using the two cell lines noted above and the parental cell line. They found that subunits of complex I and III, molecular chaperones, and a protein involved in cell cycle control were down-regulated and that inosine 5'-monophosphate dehydrogenase type 2 (IMPDH2), which is involved in nucleotide biosynthesis, was up-regulated in ρ0 cells. Retrograde proteins identified in these studies might be useful as therapeutic targets due to their roles as potential tumor suppressors or oncogenes involved in carcinogenesis.

Several investigations on other tumor mitochondrial proteome were also conducted recently. Regarding breast cancer, Strong et al [38] identified differentially expressed proteins in the mitochondria of MCF-7 human breast cancer cells that were selected for resistance to adriamycin accompanied by verapamil. Those identified proteins were mainly involved in apoptosis, heme synthesis, fatty acid oxidation, and oxidative phosphorylation. The implications of these changes in protein levels are relevant to mechanisms of drug resistance. Craven [39] compared the mitochondrial proteome in VHL (von Hippel Lindau, a tumor suppressor gene)-defective RCCs (renal cell carcinomas), which were transfected with either a control vector or wild-type VHL. That study showed that the mitochondrial protein ubiquinol cytochrome c reductase complex core protein 2 was up-regulated and a form of septin 2 was down-regulated following VHL transfection. Septin 2 was up-regulated in 12/16 RCCs. Thus, increased expression of septin 2 is a common event in RCC, and protein modification may also change the function of septin 2 in a subset of tumors. Zhao et al [40] incubated the LNCaP cell-line with sms (somatostatin)14/smsds and demonstrated that proteins with roles in apoptosis were both up-regulated (VDAC1, VDAC2) and down-regulated (PRDX2, TCTP). Sms/smsdx was believed to trigger the up-regulation of catalytic mitochondrial proteins and seemed to affect apoptosis-related proteins.

Only a few studies have reported the effects of hematological disease on the mitochondrial proteome. Yu et al [41] analyzed protein expression profiles of fractionated nuclei, mitochondria, crude endoplasmic reticulum, and cytosols of the NSC606985-induced apoptotic AML cell line NB4 cells using 2-DE combined with MALDI-TOF/TOF. They identified 90 unique deregulated proteins that contributed to multiple functional activities including DNA damage repair, chromosome assembly, and mRNA processing as well as biosynthesis, modification, and degradation of proteins. More interestingly, several oxidative stress-related proteins that were shown up-regulated were localized in mitochondria, while proteins that were up-regulated with roles in glycolysis were mainly localized in the nuclei. Their discoveries shed new insights for systematically understanding mechanisms of the camptothecin-induced apoptosis. In our previous study, we investigated mitochondrial proteome alterations in NHL Raji cells exposed to adriamycin. Our results showed that 34 proteins were down-regulated and 3 proteins up-regulated when the study group was compared with the control group. The differentially expressed proteins play roles in many cellular functions, including redox, DNA repair, cell cycle regulation, transporters and channels, and OXPHOS. Furthermore, HSP70, ABCB6, and PHB identified in this study may be closely related to chemoresistance, and this potentially serving as chemotherapeutic targets for NHL [42].

Collectively, studies on mitochondrial proteomics will further investigate into cancerous biological behavior and mechanisms of antineoplastic agents. Subsequently, improved diagnosis, and treatment methods, and new treatment targets will likely be obtained.

### Other Diseases

Mitochondrial proteomics also revealed a number of significant findings in other diseases, such as hepatopathy, placenta changes, and skeleton muscle disease.

The liver is an important organ that has an abundance of mitochondria. Ruepp [43] investigated the effects of acetaminophen (APAP) in the liver on the proteomic level and found that chaperone proteins HSP10 and HSP60 were readily decreased by half in mitochondria at different doses of APAP. The decrease of ATP synthase subunits levels and β-oxidation pathway proteins indicated a loss of energy production. Douette and coworkers [44] compared mitochondrial protein patterns in wild-type and steatosis-affected liver and identified 58 proteins exhibiting significantly different levels in these two samples. Interestingly, major proteins that regulate the generation and consumption of the acetyl-CoA pool were dramatically changed during steatosis. Furthermore, many proteins involved in the response to oxidative stress were also affected. Lee [45] assessed global protein expression profiles in term placentas from scNT (somatic cell-derived nuclear transfer)-derived and control animals. Forty-three unique proteins were identified, including such proteins play critical roles in the apoptosis signaling pathways as 14-3-3 proteins were up-regulated in scNT-derived when compared to the Annexin V in control animals group. Their results suggested that placental insufficiency in scNT-derived placentas may be due to apoptosis, induced in part by the down-regulation of 14-3-3 proteins and the up-regulation of Annexin V. De Palma [46] investigated the hypoxia-induced changes of rat skeletal muscle and indicated that proteins involved in the TCA cycle, ATP production, and electron transport are down-regulated, whereas glycolytic enzymes and deaminases involved in ATP and AMP production were up-regulated. The up-regulation of the hypoxia markers hypoxia inducible factor 1 (HIF-1α) and pyruvate dehydrogenase kinase 1 (PDK1) suggested that in vivo adaptation to hypoxia requires an active metabolic switch. Eccleston et al hypothesized that chronic exposure to a high fat diet (HFD) would modify the liver mitochondrial proteome, which might ultimately compromise mitochondrial function. Using two-dimensional isoelectric-focusing (IEF)/SDS-PAGE, 22 proteins which played roles of oxidative phosphorylation, lipid metabolism, sulfur amino acid metabolism, and chaperone proteins, showed altered levels as a consequence of the HFD. These proteomic studies were complemented by measuring mitochondrial ROS production and assessing the impact of a HFD on the levels of two key enzymes involved in maintaining tissue NO: arginase1 and endothelial nitric oxide synthase (eNOS) [47].

Alves [48] studied the influence of lifestyle on the aging alterations in skeletal muscle mitochondrial proteins with 2-DE combined MALDI-TOF/TOF. Their results confirmed that certain mitochondrial proteins, particularly those play role in the citric acid cycle and as OXPHOS components, showed increased carbonylation. The data obtained indicated that lifestyle was a key modulator for preventing the expression and functionality of aging-induced proteins in mitochiondria.

Overall, there is already considerable information regarding the important role of mitochondria in the regulation of apoptosis, energy metabolism, and electron transfer. Mitochondrial proteomics have been performed in various fields and have gained considerable achievements. Mitochondrial proteomics are currently the most popular area of subcellular proteomics being investigated.

## Challenges

Proteomic techniques are becoming more and more advanced established. However, the study of proteins is not similar to that of DNA and RNA. First, proteins have more complicated two- and three-dimensional structures, and second, proteins cannot be amplified like DNA. Protein structure can be easily altered, but cannot be easily detected if the amount is too small. As far as the mitochondrial proteome is concerned, many questions remain unresolved.

(1) *The isolation and purification of mitochondria: *At present, the well-recognized method for isolating mitochondria from tissues or cells is Taylor's classic method, which uses sucrose density gradient centrifugation [3]. However, this method requires ultracentrifugation and is time-consuming. Many efforts have been made to improve Taylor's method [49,50]. Furthermore, special kits for mitochondria isolation have been put onto the market that do not require ultracentrifugation and are more time-efficient. The purity of the mitochondria isolated by these kits has been shown to be fairly good [51].

(2) *Limitations of 2-DE: *2-DE is not good for solving many problems, such as how to remove high abundant proteins or how to isolate proteins with extreme alkalinity or acidity. With regard to mitochondria, a large proportion of the proteins have an extreme alkaline isoelectric point (pI). As a result, they are unable to be resolved by isoelectric focusing due to endo-osmotic effects upon the pH gradient [12]. In fact, some proteins have a pI that is too alkaline to be visible on typical wide range (pH 3-10) immobilized pH gradient (IPG) strips. For example, the pI of cytochrome c is 10.3 [13]. Although 2-DE is a powerful instrument, for this purpose, it still needs further improvement or replacement by other more effective techniques.

(3) *The limitation of bioinformatic tools and the mitochondrial database: *To meet the bioinformatic requirements of large-scale proteomic studies, many researchers have tried to use series-based attempts to overcome the shortcomings of single proteomic techniques. White and his colleagues use five parallel methodological approaches, ((i) peptide-centric 1-DLC, (ii) peptide-centric 2-DLC, (iii) protein-centric 1-DLC, (iv) protein-centric 2-DLC, and (v) subfractionated mitochondria) to improve the coverage of the partially annotated rabbit mitochondrial proteome prior to mass spectrometry. They found that the overall coverage of the cardiomyocyte mitochondrial proteome was improved by this parallel approach where the total number of nonredundant peptides or proteins was nearly 2 fold and more than 1.5-fold, respectively, greater than that by any single technique. They assumed that observation of proteins across multiple technologies improves the likelihood of true mitochondrial localization [52]. Furthermore, for the homologous proteins and redundant entries in the sequence database, one of the challenges in protein identification is that many peptides can be matched to several different proteins [53]. It would be helpful to use more than one database search engine when analyzing complex protein mixtures from the same raw data [54]. However, manual comparison and analysis of large database search results are laborious and time-consuming. Furthermore, most of these tools can utilize data from only a few database search engines, and currently, there are no free tools that could be used to combine protein identification results from paragon with results from other search engines. Thus, it is necessary to develop fast, accurate, and easy-to-use tools to integrate and compare protein identification results.

The insufficiency of mitochondrial databases is another problem. The first attempt to build a 2-DE database of the mitochondrial proteome was performed by Rabilloud [55]. However, that database was considered both incomplete and inefficient. Now, a large amount of new data has been added into databases such as SWISS-PROT, NCBI, MITOMAP, mtDB, hmtDB, MitoP2, MigDB, and MitoProteome [56-58]. MitoP2 is the most comprehensive database for the mitochondrial proteome and includes a more complete set of these mitochondrial proteins for human (624), mouse (615), and yeast (522) for each of these organisms respectivly [58]. However, due to the emergence of this new subject, the databases are still very insufficient and require more exploration for enrichment.

(4) *Clinical applications: *Many studies on the mitochondrial proteome have been reported, and how to utilize the findings in the clinic to gain the maximum benefits is still a large problem. Only a few of identified biomarkers have been currently used by clinicians as diagnostic and/or prognostic factors [59,60]. Novel biomarkers identified by proteomics can be developed for increased precision in diagnosis, therapy sensitivity, progression, and prognosis evaluation of disease. Information on bioinformatics obtained from proteomic analyses is still scarce. These challenges have not been overcome by the existing methods and they have become a limitation to further advancement. Understanding how mitochondrial proteins function together in pathways and complexes is still a significant challenge. Many biomarkers found in proteomic studies were conformed to be involved in various mitochondrial-associated signaling pathways, including apoptosis, cell cycle, and DNA repair [61,62]. Many validation tests using methods such as RNAi, protein-protein interaction mapping, and computational predictions should be linked to the future investigations [63,64].

Standardizing these current proteomic experiment in terms of sample collection, storage and processing as well as bio-informatics and statistical analysis between various centers is serious necessary [65]. Future perspectives will focus on the clinical applications of these biomarkers to improve diagnostic accuracy and prognostic precision. Thus, more intimate, repeatable, and verifiable experiments are eagerly awaited.

## Conclusions

The emergence of comparative proteomics enables us to investigate the mitochondrial proteome in a more comprehensive and effective manner. The results of mitochondrial proteomics provide a better understanding of the pathogenesis associated with mitochondria and generate promising therapeutic targets. However, these novel findings are most unlikely to completely reflect the true state of mitochondria because some biological information may be lost or altered during the course that mitochondria are isolated from the cell. Moreover, the mitochondrial proteome alterations in animal models may differ from those of human. Therefore, more efforts are needed to look at the validation across species carefully. Validation and utilization of clinically associated proteomic biomarkers would be helpful to the diagnosis, effective treatment, and prognosis evaluation of mitochondria-mediated diseases. Thus, the formulation of personalized medicine may become a reality in the future. This is an open-ended exploration, and more achievements are anticipated in the near future.

## Competing interests

The authors declare that they have no competing interests.

## Authors' contributions

Both authors participated in drafting and editing the manuscript. Both authors read and apporoved the final manuscript.
